# Potential Utility of Combined Urine Lipocalin‑2 and Copper Test in Diagnosing Acute Pyelonephritis or Cystitis

**DOI:** 10.1021/acsomega.5c05922

**Published:** 2025-09-17

**Authors:** Jan Hrbáček, Tomáš Pluháček, Jiří Novák, Andrea Palyzová, Dominika Luptáková, Jiří Houšt, David A. Stevens, Radim Dobiáš, Roman Zachoval, Vladimír Havlíček

**Affiliations:** † Department of Urology, Thomayer University Hospital and Third Faculty of Medicine, Charles University, Vídeňská 800, Prague 140 59, Czechia; ‡ Department of Urology, 48239 Bulovka University Hospital, Budínova 67/2, Prague 180 81, Czechia; § Institute of Microbiology of the Czech Academy of Sciences, Vídeňská 1083, Prague 142 00, Czechia; ∥ Department of Analytical Chemistry, Faculty of Science, Palacký University, 17. listopadu 12, Olomouc 771 46, Czechia; ⊥ Biomedicine Research Centre of the Slovak Academy of Sciences, Institute of Virology, Dúbravská Cesta 9, Bratislava 84505, Slovakia; # Division of Infectious Diseases and Geographic Medicine, Stanford University School of Medicine, Stanford, California 94305, United States of America; ¶ Department of Bacteriology and Mycology, National Reference Laboratory for Mycological Diagnostics, Public Health Institute in Ostrava, Partyzánské náměstí 2633/7, Ostrava 702 00, Czechia; ∇ Institute of Laboratory Medicine, Faculty of Medicine, University of Ostrava, Syllabova 19, Ostrava 703 00, Czechia

## Abstract

The noninvasive differentiation of acute pyelonephritis (AP) and complicated urinary tract infections (cUTI), from acute cystitis (AC), represents an important diagnostic challenge in urology and nephrology. In a study of 95 adults, urine from patients with AP (*n* = 17), cUTI (*n* = 6), AC (*n* = 28), and controls (*n* = 44) was examined using a multidisciplinary approach: enzyme-linked immunosorbent assays for quantitation of acute phase proteins ceruloplasmin (Cp), pentraxin-3 (Ptx3), and lipocalin-2 (Lcn2); inductively coupled plasma mass spectrometry for determination of total copper (Cu), zinc (Zn), and iron (Fe) concentrations; and liquid chromatography coupled to electrospray ionization MS (LC–MS) for identification and quantitation of bacterial metallophores. Markedly elevated levels of human Cp, Ptx3, and Lcn2, all normalized to urine creatinine (Cr), in AC, cUTI, and AP patients compared to controls identified ongoing urinary tract infection without further differentiation between AC and AP. On the contrary, total urine copper normalized to Cr (Cu/Cr index) significantly differentiated AP from AC (*p* = 0.0209), as well as from the controls. Bacterial metallophores aerobactin and yersiniabactin, but not enterobactin, were indicators of AP, cUTI, and AC caused by *Enterobacteriaceae*. Importantly, the newly proposed combined test based on the quantitation of Lcn2 normalized to Cr (Lcn2/Cr) and Cu/Cr could noninvasively differentiate AP and cUTIs from AC with a diagnostic accuracy of 78% sensitivity and 65% specificity. The combination of characteristic elemental and molecular biomarkers may represent a future research direction with the potential to improve diagnostics of UTIs.

## Graphical abstract

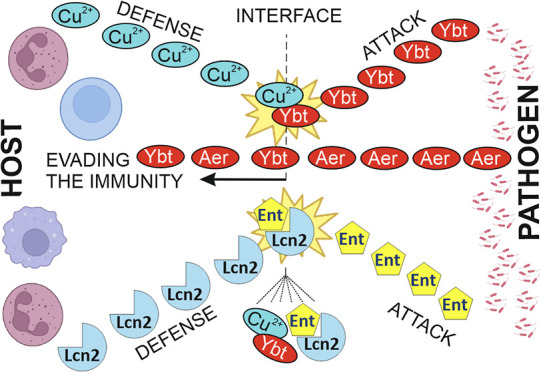

## Introduction

Urinary tract infections (UTIs) can range from mild to life-threatening, affecting over 150 million people worldwide annually with a higher incidence in women.^1^ It is estimated that half of all women will experience at least one UTI during their lifetime, with up to 50% of them encountering a recurrent UTI within six months.^2^ Consultations for UTIs account for 1–6% of all medical visits.^3^ The majority of UTIs are caused by uropathogenic *Escherichia coli*, followed in prevalence by *Enterococcus* spp., *Klebsiella* spp., *Pseudomonas aeruginosa*, and *Proteus* spp.^4^

The tug-of-war over essential elements between hosts and invading pathogens represents a key mechanism in UTI development.^5^ Uropathogens obtain essential metallic nutrients in metal-limited settings via discharge and subsequent absorption of scavenging chelators, known as metallophores (or siderophores when capturing iron). Extraintestinal pathogenic *E. coli* selectively induce yersiniabactin (Ybt) production in extreme (low or toxic) extracellular cupric cation concentrations.^6^ Furthermore, Ybt safeguards intracellular pathogens from the respiratory burst within Cu-containing phagosomes by forming Cu­(II) complexes that imitate superoxide dismutases.^7^ Another siderophore secreted by uropathogens is aerobactin (Aer), which has a higher iron association constant (log *K* = 28) than host transferrin (log *K* = 22). It is considered to be a contributing factor to the hypervirulence observed in specific *Klebsiella pneumoniae* phenotypes as documented in animal models.^8^

The capacity to elicit an immune response may be an attribute of siderophores, leading to the overexpression of host proteinaceous lipocalins (e.g., Lcn1 and Lcn2), molecules that mobilize immune cells.^9^ The typical serum concentrations of Lcn2 (approximately 1–3 nM) can increase to approximately 0.1 mM during an infection.^10^ In healthy adults or children urine, median urine Lcn2 levels are typically very low at ∼0.16 or 0.8 nM, respectively.^11,12^ In a recent meta-analysis, UTI-associated Lcn2 levels frequently ranged from 50 to several hundreds of ng/mL, especially in acute pyelonephritis (AP), i.e., from ∼2 to ∼13 nM.^13^

Another key player in the competition for nutrients is ceruloplasmin (Cp), a mammalian ferroxidase enzyme and an extracellular Cu carrier that is overexpressed in response to microbial infection.^14^ Pentraxin-3 (Ptx3) is an acute phase protein host factor that can help differentiate bacterial diseases from invasive pulmonary fungal infections.^15^ This study aimed to investigate an innovative diagnostic approach with particular emphasis on the discrimination between acute pyelonephritis and complicated urinary tract infections (AP-cUTI) and acute cystitis (AC) based on a combined analysis of acute phase proteins, bacterial metallophores, and metal pools in urine as diagnostic markers of UTIs.

## Results

### Clinical and Demographic Characteristics of the Population

A total of 47 female and 48 male subjects with a mean age of 47.1 years (standard deviation [SD] 18.7) and 61.7 years (SD 16.3), respectively, were included in the study (Figure 1A–C). Their assignments to controls (Ctrl, *n* = 44), AC (*n* = 28), cUTI (*n* = 6), and AP (*n* = 17) groups, including descriptive statistics, are listed in Table 1. Symptoms of AP and cUTI groups (Clinical Study Design in Materials and Methods) persisted for a mean of 4.5 days (SD 3.1); mean peripheral white blood cell count and C-reactive protein levels were 16.1 mg/L (SD 4.6) and 184.4 mg/L (SD 71.6), respectively. Flank pain or costovertebral tenderness represented AP-localizing symptoms. In the AP cohort (Table S1), *E. coli* variants were the most frequently recovered pathogens by culture (*n* = 8, 47%). The next most commonly encountered pathogens were *Klebsiella* spp. (*n* = 5, 29%), *Staphylococcus aureus* (*n* = 1, 6%), and *Proteus mirabilis* (*n* = 1, 6%). Two AP patients tested were culture-negative (12%). In the AC group, the most prevalent bacterial pathogens were *E. coli* spp. (*n* = 19, 68%) and then *Staphylococcus* spp. (*n* = 5, 18%), *Candida albicans* (*n* = 1, 3.5%), *Serratia marcescens* (*n* = 1, 3.5%), *Klebsiella* spp. (*n* = 1, 3.5%), and *P. aeruginosa* (*n* = 1, 3.5%).

**Figure 1:**
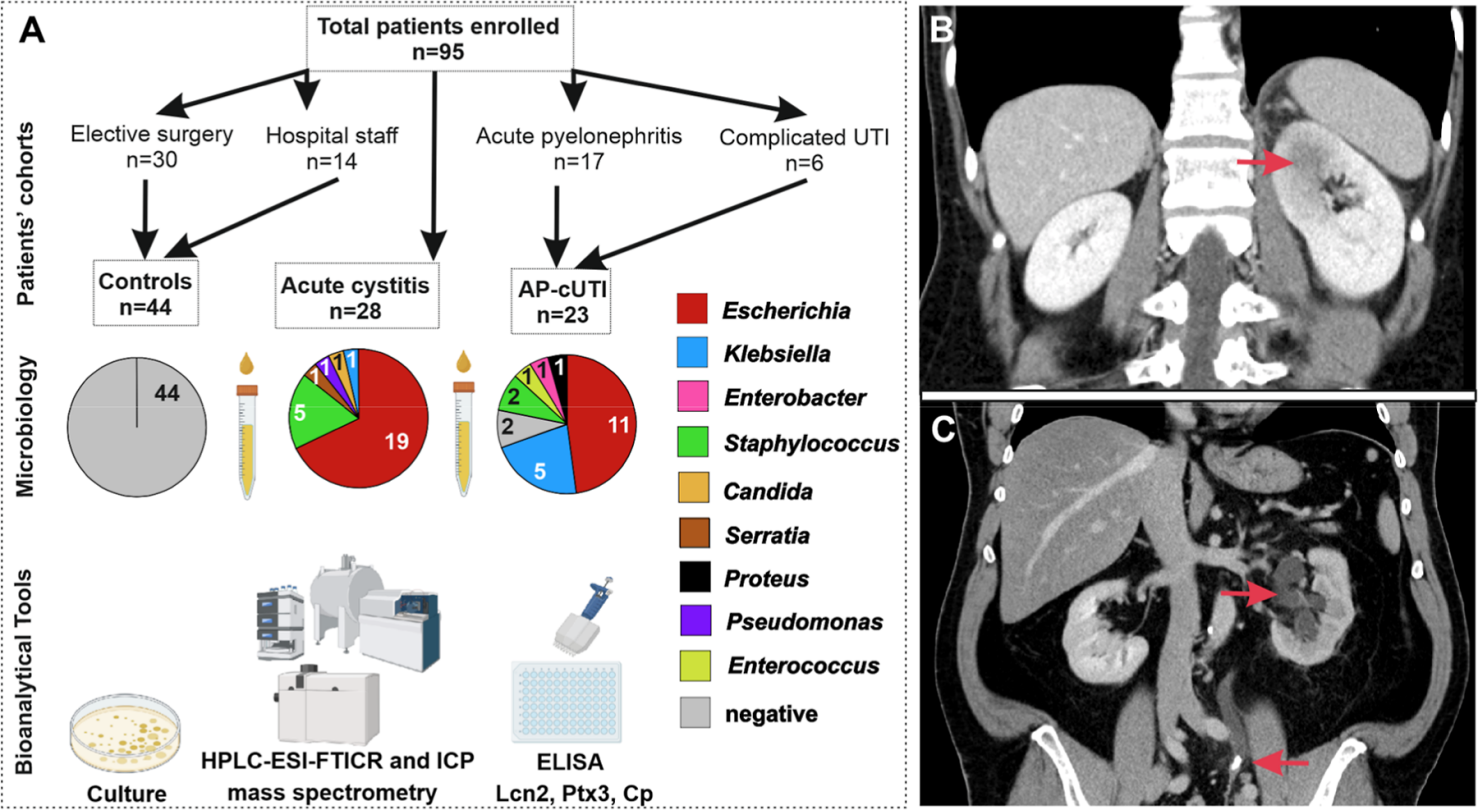
Study design, uropathogens, and urine sample processing workflow. All bioanalytical tools shown in (A) were applied to urine samples from all cohorts. (B) Computed tomography scan of a 43 year-old female patient (No. 6 in Table S1) with focal pyelonephritis of the left kidney indicated with a red arrowhead in the coronal section. (C) Computed tomography scan of a 60 year-old male patient (No. 1 in Table S1) with obstructive pyelonephritis of the left kidney, dilated collecting system (top arrowhead), and ureteric stone causing obstruction (bottom arrowhead). HPLC-ESI-FTICR-MS, high-performance liquid chromatography electrospray ionization Fourier transformed mass spectrometry; ICP-MS, inductively coupled plasma mass spectrometry; ELISA, enzyme-linked immunosorbent assay; Lcn2, lipocalin-2; Cp, ceruloplasmin; AP, acute pyelonephritis; and cUTI, complicated UTI. The image was generated in part using BioRender.com (https://BioRender.com/cyxejer).

**Table 1:** Biomarker Frequencies and Population Study^a^

study: NCT05674032	groups	Ctrl	AC	cUTI	AP
number of patients (%)	total	44	28	6	17
	male	37 (84)	3 (11)	4 (67)	3 (18)
	female	7 (16)	25 (89)	2 (33)	14 (82)
age [years, mean ± SD]	total	59.0 ± 17.6	43.3 ± 20.8	69.7 ± 9.8	46.8 ± 16.6
	male	59.5 ± 17.7	66.0 ± 25.1	70.8 ± 11.9	55.0 ± 13.2
	female	56.4 ± 18.2	40.5 ± 19.1	67.5 ± 6.36	45.0 ± 17.1
bacteria recovered by culture [frequency (%)]	*E. coli*	0	19 (68)	3 (50)	8 (47)
	*Kleb* spp	0	1 (3)	0 (0)	5 (29)
	other	0	8 (29)	3 (50)	2 (12)
	none	44 (100)	0	0 (0)	2 (12)
metal indices [μg/mmol, median (IQR)]	Cu/Cr	1.6 (1.6–2.2)	2.3 (1.6–3.9)	4.0 (2.3–7.6)	5.2 (2.2–12.8)
	Fe/Cr	14.8 (5.5–57.2)	18.1 (5.5–114.0)	14.1 (5.2–24.1)	15.2 (8.1–127.5)
	Zn/Cr	42.7 (28.8–69.3)	63.9 (29.1–89.1)	78.0 (53.8–105.6)	57.5 (41.4–148.5)
microbial metallophore [frequency (%)]	Ybt	0	4 (14)	2 (33)	1 (6)
	Aer	0	1 (4)	2 (33)	4 (24)
	Pch	0	0	1 (17)	0 (0)
host proteins [μg/mmol, median (IQR)]	Cp/Cr	0.7 (0.7–3.2)	22.0 (8.9–52.5)	43.3 (9.4–385.2)	45.1 (18.5–105.2)
	Lcn2/Cr	0.1 (0.1–0.1)	0.5 (0.3–0.7)	0.7 (0.4–10.8)	1.6 (0.5–2.7)
	Ptx3/Cr	2.4 (2.4–7.1)	24.9 (4.3–53.1)	32.0 (5.8–420.8)	32.4 (19.3–61.4)

a AP and cUTI, inpatients admitted for the treatment of AP and complicated UTI, respectively; AC, outpatients seeking treatment for uncomplicated AC; Ctrl, inpatients admitted to the hospital for elective surgery without UTI signs or symptoms; Cr, creatinine; SD, standard deviation; IQR, interquartile range; Ybt, yersiniabactin; Aer, aerobactin; Cp, ceruloplasmin; Lcn2, lipocalin 2; Ptx3, Pentraxin 3; and Pch, pyochelin; *Kleb.*, *Klebsiella*.

### Host Protein and Total Copper Urine Metal Levels in UTI Patients and Controls

Human Ptx3, Lcn2, Cp, and total copper creatine-adjusted indices (Ptx3/Cr, Lcn2/Cr, Cp/Cr, and Cu/Cr) levels were significantly elevated in urine samples of AC, cUTI, and AP patients compared to Ctrls, and they showed the same increasing concentration trend from AC to AP (Figure 2A–C). Median Lcn2/Cr indices with interquartile range (IQR) were 0.1 (0.1–0.1), 0.5 (0.3–0.7), 0.7 (0.4–10.8), and 1.6 (0.5–2.7) μg/mmol in Ctrl, AC, cUTI, and AP patient groups, respectively (Table 1). Median Cu/Cr indices and IQR were 1.6 (1.6–2.2), 2.3 (1.6–3.9), 4.0 (2.23–7.6), and 5.2 (2.2–12.8) μg/mmol in Ctrl, AC, cUTI, and AP patient groups, respectively (Table 1). Of note, total urinary Cu/Cr indices discriminated between AP and AC (*p* < 0.0209, Figure 2D).

**Figure 2:**
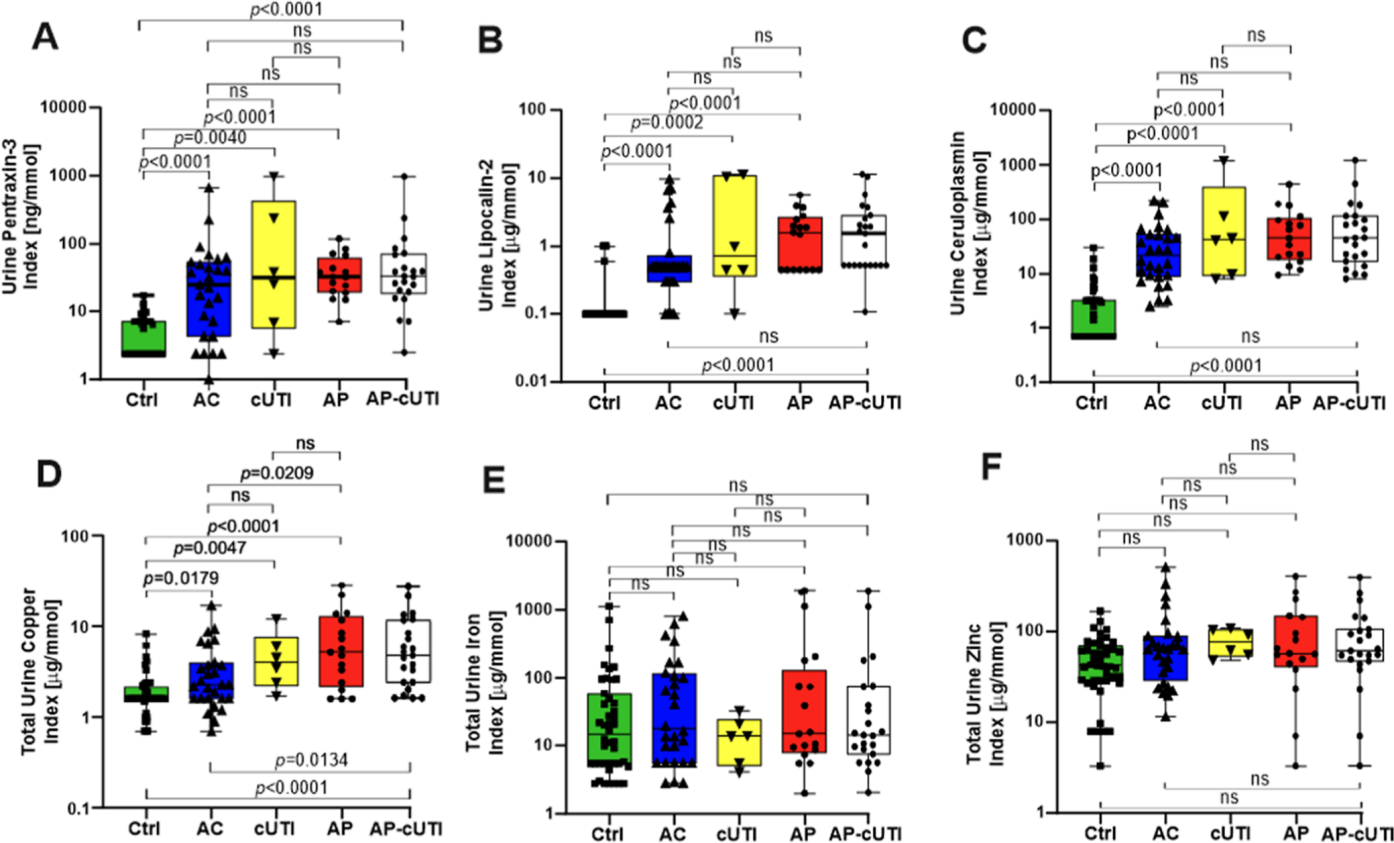
Individual biomarker indices adjusted for urine creatinine. (A–C) Host pentraxin-3, lipocalin-2, and ceruloplasmin were significantly overexpressed in AC, cUTI, AP, and AP-cUTI compared to controls (Ctrls) but not in AP vs AC, AP vs cUTI, or AP-cUTI vs AC. (D) Copper levels were higher in UTI (AC, cUTI, AP, and AP-cUTI) patients versus Ctrls and in AP (or AP-cUTI) patients versus AC patients. AP and cUTI patients could not be distinguished. (E,F) Total zinc and iron levels were not significantly different among groups.

Similar significance between AC and AP-cUTI, *p* < 0.0134, was obtained, when AP and cUTIs were merged into one group (AP-cUTI). For purpose of analysis, the study groups AP and cUTI were kept merged, and further results were presented using the combined AP-cUTI patient group without impacting the statistical significance versus Ctrl or AC groups.

Correlation between measured proteins was determined using Spearman’s correlation coefficients (Table 2). Moderate correlation between Ptx3/Cr and Cp/Cr (*r* = 0.60), and between Ptx3/Cr and Lcn2/Cr (*r* = 0.70), was demonstrated. Statistically significant (*p* ≤ 0.05) correlation was found between Cp/Cr and Lcn2/Cr (*r* = 0.71).

**Table 2:** Spearman *r* Correlation Coefficients^a^

variable	Cu/Cr	Fe/Cr	Zn/Cr	Cp/Cr	Lcn2/Cr
Fe/Cr	0.3735				
**Zn/Cr**	0.2224	0.0885			
**Cp/Cr**	**0.4788**	0.1545	0.1948		
**Lcn2/Cr**	**0.4958**	0.1846	0.1916	**0.7065**	
**Ptx3/Cr**	**0.4564**	0.2026	0.2527	**0.5982**	**0.6981**

a Statistically significant (*p* ≤ 0.05), moderate (*r* > 0.3), and strong correlations (*r* > 0.7) are highlighted in red. Cu/Cr: total copper index; Fe/Cr: total iron index; Zn/Cr: total zinc index; Cp/Cr: ceruloplasmin index; Lcn2/Cr: lipocalin-2 index; and Ptx3/Cr: pentraxin-3 index.

With >90% specificity, Cp/Cr, Ptx3/Cr, and Lcn2/Cr levels with 100%, 86%, and 96% sensitivities, respectively, classified subjects as belonging to the AP-cUTI group versus Ctrls (Table S2). With the same >90% specificity, the protein indices provided 79%, 67%, and 86% sensitivities for the discrimination between the AC group and Ctrls. Lcn2/Cr provided the highest sensitivity in both groups. In addition to the separate comparison of AP-cUTI or AC to Ctrls, a combined (AP-cUTI + AC) comparison against the Ctrls was performed. Lcn2/Cr remained the best performing biomarker with 90% sensitivity and 98% specificity at 0.1 μg/mmol cutoff for the discrimination between healthy Ctrls and patients with UTI of any type (Table S2). Note that total zinc and iron levels were not significantly different among groups (Figure 2E, F).

### Frequency and Value of Detection of Bacterial Metallophores in UTI Patients and Controls

No microbial metallophores were recorded in the Ctrls (Table S3). Active bacterial proliferation, assumed by metallophore secretion, was observed in 18% of the patients diagnosed with AC (5/28) (Table S4) and in 39% of AP-cUTI patients (9/23). In the AP-cUTI group, Aer (*n* = 6) was the prevailing metallophore with creatinine-adjusted indices fluctuating in patients from 2.4 to 53.7 μg/mmol (Table S1). Aer was secreted by *E. coli* spp. (*n* = 4), *Klebsiella* spp. (*n* = 1), and *Enterococcus* spp. (*n* = 1). Bacterial Ybt was consistently present in both the AP-cUTI (*n* = 3, Table S1) and AC (*n* = 4, Table S4) groups, and chelated Fe or Cu (Table S1), but was not detected in Ctrls (Table S3). The urine cupric yersiniabactin (CuYbt/Cr) index reached 10.4 μg/mmol (Figure S1) in the AP-cUTI group. The presence of CuYbt could reflect a response of some bacterial pathogens to excessive copper concentrations at the site of inflammation by passivation.^16^ Serial day sampling in the AP-cUTI cohort revealed prompt metallophore attenuation in urine samples from all patients after antibiotic treatment was initiated (data not shown). Irrespective of the low nanogram limit of detection (Figure S2), no free bacterial enterobactin (Ent) was detected in any urine sample involved in this study, nor metallophores of *Staphylococcus* and *Candida* spp.

### Combination Biomarker Test for the Differentiation between AP-cUTI and AC

The AP and cUTI groups could be distinguished from the AC group using a two-step diagnostic approach. In the first step, patients with disease (AP and cUTI) were defined as those with an Lcn2/Cr index >0.45 μg/mmol (Table 2). In the second step, ambiguous patient cases with an Lcn2/Cr index of 0.45 μg/mmol (DET) were sorted by elevated urinary Cu/Cr index.^17,18^ Patients with Cu/Cr ≥ 4.8 μg/mmol (median for AP + cUTI groups) were classified as belonging to the AP and cUTI group, while patients with Cu/Cr < 4.8 μg/mmol were classified as the AC group. The combination of both biomarkers (Lcn2/Cr and Cu/Cr) in one comparison resulted in a sensitivity of 78% and a specificity of 65% (Figure 3A,B). The addition of bacterial metallophore detection as a third component in a combination test provided an improvement in sensitivity (87%) to the detriment of specificity (57%).

**Figure 3:**
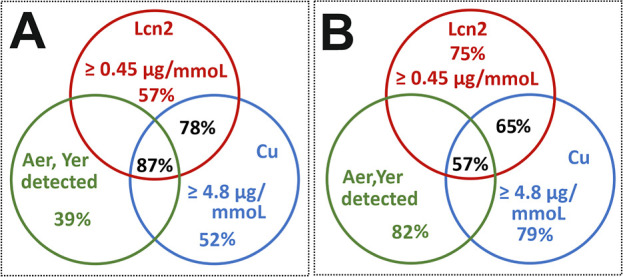
Combined Lcn2/Cu test performance in the UTIs. Sensitivity (A) and specificity (B) performances were graphically expressed according to Table S5 data. Lcn2, lipocalin-2; Ent, enterobactin; Aer, aerobactin; Yer, yersiniabactin; ELISA, enzyme-linked immunosorbent assay (red circles); and MS, mass spectrometry (green and blue circles).

## Discussion

Copper is a host effector that is mobilized into the urine during UTI to interfere with bacterial growth and urine Cu concentrations can rise to 0.5 μM during an infection episode.^17^ In this study, Cu/Cr index was the only discriminatory marker expressed differentially in AP-cUTI and AC patients. In the host, our probable explanation is that the volume of infected uroepithelial cells is higher in the AP-cUTI, compared with the AC group. Pathogenic bacteria negatively regulate excessive Cu concentrations via Ybt extracellular production, which may contribute to their resistance.^18^ On the contrary, the Fe/Cr and Zn/Cr indices did not reveal notable differences between the study groups (Figure 2E,F). The total copper level can be accessed by either inductively coupled plasma mass spectrometry (ICP–MS) or ICP optical emission spectrometry. While ICP instrumentation is costly, spectrophotometric quantitation is cheaper and is often available in clinical laboratories. This easier and cheaper option represents another strong argument for the future use of the combined Lcn2/Cu assay in future studies on larger cohorts.

In the present study, metallophores were detected in 5/17 (29%) AP and 4/6 (67%) cUTI patients (Table S1), suggesting potential clinical usefulness in either situation. Unfortunately, they could not distinguish between patients with AP-cUTI and those with AC which would be an important clinical advancement. Bacterial Ybt was consistently present in both the AP-cUTI and AC groups and chelated Fe or Cu (Table S1). In the AC cohort, a single Aer-finding was recorded with a 22 year female outpatient with hemolytic *E. coli* infection (Table S4). Although Aer (ferri, desferri, and anhydro desferrri) reached an FeAer/Cr index of 5.031 μg/mmol in this patient, we speculate that considerable host fitness combined with a high Lcn2 response may have protected this patient from the criteria for hospitalization. Lcn2 is an Ent scavenger and is overexpressed by immunocompetent hosts during infection episodes.^20^ In this study, we assume that bacterial Ent, if produced, was entirely bound to Lcn2. As a result, no free Ent was detected in any patient sample. As Lcn2 is also increased in chronic or acute renal failure, coronary artery disease, atrial fibrillation, and Wilson disease,^11,21,22^ their frequency needs to be strictly controlled between groups in any follow-up studies. On the contrary, urinary Lcn2 deficiency was recorded in recurrent UTIs.^12^

Unlike Lcn2, which is an Ent chelator, the role of other host proteins in diagnosing UTIs has not been confirmed (Table 1). No correlations were found between the total Fe and Lcn2. Similarly, there was no correlation between clinically used inflammatory markers (white blood cell count and C-reactive protein) and the presence of bacterial metallophores (data not shown).

### Strengths and Limitations

The substrate for our assays, urine, is an easily and conveniently obtained abundant specimen for study and facilitates serial observations, should that prove advantageous (such as following the course of illness and efficacy of treatment). A metallophore approach can be more sensitive and specific than conventional approaches as demonstrated in the diagnostics of pulmonary infections.^23^ It is also faster, since it is a culture-free approach with mass spectrometry analysis taking approximately 1 h. In the present study, benchmarking metallophore approach against the detection by culture, metallophores of *E. coli*, *K. pneumoniae*, *Enterobacter*, and *Enterococcus* spp. resulted in a sensitivity of 75% in the AP-cUTI group (9/12). Two false-negative microbial cultivation cases were noted in the AP-cUTI group, and confusing microbiological results are also expected for polymicrobial infections. One finding of note was *Enterobacter* spp. recovered from patient 5 (cUTI), from whom pyochelin enantiomers, characteristic of *P. aeruginosa*, were recorded by LC–MS.

This study has some limitations. Having determined clinically useful indices in this study, the indices must be confirmed prospectively in substantial patient populations to provide firmer conclusions.^24^ Following the Standards for Reporting Diagnostic Accuracy guideline^25^ and considering the expected prevalence of UTIs is 40%, then the 60 participants required for the estimation of sensitivity are 40% of the study sample, resulting in a total number of participants of at least 150.^26^

In the AC and Ctrl groups, we recorded similar Cu/Cr medians in both female and male groups. Although the higher Cu/Cr indices in the male AP group could be attributed to larger tissue volumes in males,^27^ this conclusion requires a larger sample size for confirmation. Second, seven patients in the AP-cUTI group received initial antibiotic therapy prior to urine collection (amoxicillin-clavulanate (*n* = 3), cefuroxime (*n* = 2), fosfomycin trometamol (*n* = 1), and ciprofloxacin (*n* = 1)), all of whom had bacterial pathogens cultured despite previous administration of antibiotics, and two of these tested positive on bacterial metallophore secretion. Adequate antibiotic treatment could eliminate the pathogen as well as its metallophores, whereas inadequate therapy (due to resistance, underdosing, etc.) likely does not.

## Conclusions

We explored three proteinaceous biomarkers that exhibited high diagnostic accuracies for UTIs (AP, cUTI, and AC) versus Ctrls: Ptx3/Cr, Lcn2/Cr, and Cp/Cr. The Lcn2/Cr index distinguished patients with UTIs from those without. Concurrently, we explored three metal levels in the patient’s urine: Cu/Cr, Fe/Cr, and Zn/Cr. Total Fe and Zn loads would not discriminate between those of healthy Ctrls and patients with UTIs. Importantly, the Cu/Cr index distinguished AC from the AP-cUTI cohort. Our data support the hypothesis that the total Cu/Cr may correlate with the volume of damaged tissue, enabling us to distinguish between AC and AP-cTUI. As a result, we suggested a two-step test based on creatinine-normalized urine Lcn2 levels and total urine Cu levels. This noninvasive, two-step assay demonstrated 78% sensitivity and 65% specificity.

## Materials and Methods

### Ethics Statement

The study NCT05674032, entitled “Bacterial Metallophores in the Diagnosis of AP, was registered at ClinicalTrials.gov. It received prior approval from the Ethics Committee of the Institute for Clinical and Experimental Medicine and the Thomayer University Hospital (No. 12205/22), and informed consent documents were acquired from all study participants. Throughout the study, all hospital and research staff followed the Good Clinical Practice guidelines outlined in the Declaration of Helsinki in 2013 and general guidelines 86/609/EEC and 200/54/EC 16 protecting the European Community from the handling of potentially infectious materials.

### Clinical Study Design

Patient population for this prospective observational study was recruited from patients of the Department of Urology, Thomayer University Hospital in Prague, Czech Republic between June 2020 and January 2023. The trial design is based on four groups of adult subjects of both sexes (Figure 1) with the following inclusion criteria:^28,29^ (1) AP or complicated UTI (cUTI) patients defined as having at least two of systemic signs and symptoms (chills, rigors, fever above 38.0 °C, nausea or vomiting, dysuria, urgency or frequency, lower abdominal pain, acute flank pain or costovertebral angle tenderness, and peripheral white blood cell count >12,000/mm^3^) AND pyuria on microscopic urine examination (>10 white blood cells/mm^3^ in unspun urine). The main discriminatory feature of AP as opposed to c-UTI is the localizing symptom of flank pain and costovertebral angle tenderness. Three patients in AP or cUTI groups had a foreign body in their urinary tracts, and some type of urinary tract obstruction was detected in seven. (2) AC defined as the presence of lower urinary tract symptoms (dysuria, urgency, and frequency) AND pyuria as defined above, in the absence of systemic signs and symptoms of infection and without any known anatomical or functional abnormality of the urinary tract and (3) Ctrl group of patients treated for a noninfectious diagnosis and healthy volunteers, without signs or symptoms of a UTI and with negative culture results.

Urine samples from all patients were stored at 4 °C and processed within 12 h of collection. Two aliquots of the same sample were sent for urine culture and frozen at −20 °C until further analysis for urine Cr, Ptx3, Cp, and Lcn2 by ELISA, metals by ICP–MS, and bacterial metallophores by LC-MS-based infection metallomics.^5^ All urinary biomarkers were normalized to the urinary Cr concentration to obtain index values expressed in μg/mmol.^23^ The urine Cr concentration was determined using an Atellica CH Analyzer (Siemens, Germany) in an Enzymatic Creatinine_3 (ECre3) assay optimized for a 0.177–21.658 mmol/L working range.

### Determination of Urine Total Cu, Fe, and Zn Levels

Urine aliquots (0.5 mL) were subjected to digestion using 1 mL of concentrated nitric acid in an Ultrawave microwave unit (Milestone, Italy) according to the biological sample digestion method provided by the vendor. Subsequently, the digested samples were transferred into a 10 mL volumetric flask filled with ultrapure water, mixed, and analyzed by the ORS-ICP-MS 7700x (Agilent Technologies) instrument, using a helium mode to eliminate spectral interferences on ^56^Fe, ^63^Cu, ^66^Zn, and ^89^Y (internal standard) isotopes. The quantitation was performed using an eight-point external calibration (Figure S2). The total metal concentration levels (μg/L) were normalized to urine Cr and indexed values are reported in Tables S1, S3, and S4 (μg/mmol).

### LC–MS Analysis of Metallophores

Metallophore quantification was conducted using a urine-matched calibration standards^5,23^ according to a detailed procedure reported in Figure S2. The extracted samples were separated using a Dionex UltiMate 3000 UHPLC liquid chromatograph system (Thermo Fisher Scientific, MA, USA) connected to a 12T solariX Fourier-Transform Ion Cyclotron Resonance mass spectrometer (Bruker Daltonics, MA, USA). One microliter of each sample was injected into a preheated (40 °C) Acquity HSS T3 C18 analytical column (1.8 μm, 1.0 × 150 mm; Waters, MA, USA) for analysis. The mass spectrometry data were processed with DataAnalysis 6.0 (Bruker Daltonics, MA, USA) and evaluated with CycloBranch 2.1.35 software.^30^ The parameters of LC–MS analysis are described in the Supporting Information, “Sample Preparation and LC–MS Analysis” section. The quantitative data of all patients’ cohorts are depicted in Tables S1, S3, and S4.

### Quantitation of Ceruloplasmin, Pentraxin-3, and Lipocalin-2

The human Lcn2 and Cp ELISA kits were purchased from RayBiotech (Peachtree Corners, GA, USA) and Assaypro (St. Charles, MO, USA), respectively, and utilized in accordance with the manufacturer’s instructions. The absorbance of Lcn2 and Cp at 450 nm was recorded using the SPECTRAmax PLUS384 well plate reader from Molecular Devices (St. Charles, MO, USA). Urine samples were analyzed in duplicates. Urine Ptx3 concentrations were measured using an ELISA kit from BioVendor (Brno, Czechia).

### Statistical Analysis

The ICP–MS, ELISA, and ESI-MS data were statistically analyzed using GraphPad Prism 10.2.3 (GraphPad, San Diego, California, USA) and tested for Gaussian distribution using the D’Agostino and Pearson normality tests. Nonparametric ANOVA (Kruskal–Wallis) and uncorrected Dunn’s posthoc multiple comparison tests were employed in all cohorts. For statistical tests, values between the LOQ and LOD (i.e., detected, DET), and below the LOD (i.e., not detected, ND), the method LOQ and LOD values were used, respectively. The strength of correlations among biomarkers was assessed using Spearman’s coefficients (Table 2).

## Supplementary Material



## Data Availability

The raw mass spectrometry data, https://hdl.handle.net/11104/0364645, can be viewed using the CycloBranch software, https://ms.biomed.cas.cz/cyclobranch/.

## Notes

Yersiniabactin profiling in *Klebsiella* spp.; calibration curves of Ptx3, Lcn2, Cp, total Fe, total Zn, total Cu, Fe-Aer, Ent, Fe-Ybt, and Pch; cohort inpatients admitted for the treatment of AP and cUTI; diagnostic accuracy of single level tests; control cohort inpatients admitted for elective surgery and healthy volunteers; cohort outpatients seeking treatment for AC; and diagnostic accuracy in a combination test (PDF)
