# Supplementary material for: Potential Utility of Combined Urine Lipocalin‑2 and Copper Test in Diagnosing Acute Pyelonephritis or Cystitis
Source: ACS Omega. 2025 Sep 17;10(38):44291–7. doi: 10.1021/acsomega.5c05922 (PMC12489698; doi:10.1021/acsomega.5c05922)
Supplement: Supplementary file 1 [file ao5c05922_si_001.pdf]

SUPPORTING INFORMATION

Potential Utility of Combined Urine Lipocalin-2 and Copper Test in Diagnosing Acute Pyelonephritis or Cystitis

Jan Hrbáček[1,2], Tomáš Pluháček[3,4], Jiří Novák[3], Andrea Palyzová[3], Dominika Luptáková[3,5], Jiří Houšt[3,4], David A. Stevens[6], Radim Dobiáš[7,8], Roman Zachoval[1], and Vladimír Havlíček[3,4,]*

[1]Department of Urology, Thomayer University Hospital and 3rd Faculty of Medicine, Charles University, Vídeňská 800, Prague 140 59, Czechia; [2]Department of Urology, Bulovka University Hospital, Budínova 67/2, Prague 180 81, Czechia; [3]Institute of Microbiology of the Czech Academy of Sciences, Vídeňská 1083, Prague 142 00, Czechia; [4]Department of Analytical Chemistry, Faculty of Science, Palacký University, 17. listopadu 12, Olomouc 771 46, Czechia; [5]Biomedicine Research Centre of the Slovak Academy of Sciences, Institute of Virology, Dúbravská Cesta 9, Bratislava 84505, Slovakia; [6]Division of Infectious Diseases and Geographic Medicine, Stanford University School of Medicine, Stanford, CA 94305, United States of America

[7]Department of Bacteriology and Mycology, National Reference Laboratory for Mycological Diagnostics, Public Health Institute in Ostrava, Partyzánské náměstí 2633/7, Ostrava 702 00, Czechia; [8]Institute of Laboratory Medicine, Faculty of Medicine, University of Ostrava, Syllabova 19, Ostrava 703 00, Czechia

*Corresponding author: vlhavlic@biomed.cas.cz

Table of Contents:

**Figure/Table**	Page
**Figure S1.** Yersiniabactin profiling in *Klebsiella* spp.	S2
**Figure S2.** Calibration curves of Ptx3, Lcn2, Cp, total Fe, total Zn, total Cu, Fe-Aer, Ent, Fe-Ybt, and Pch. Determination of total copper, iron and zinc levels in urine by ICP-MS. Sample Preparation and LC-MS Analysis.	S3
**Table S1.** Cohort inpatients admitted for the treatment of AP and cUTI.	S5
**Table S2.** Diagnostic accuracy of single level tests.	S6
**Table S3**. Control cohort inpatients admitted for elective surgery and healthy volunteers.	S7
**Table S4.** Cohort outpatients seeking treatment for AC.	S9
**Table S5.** Diagnostic accuracy in a combination test.	S10

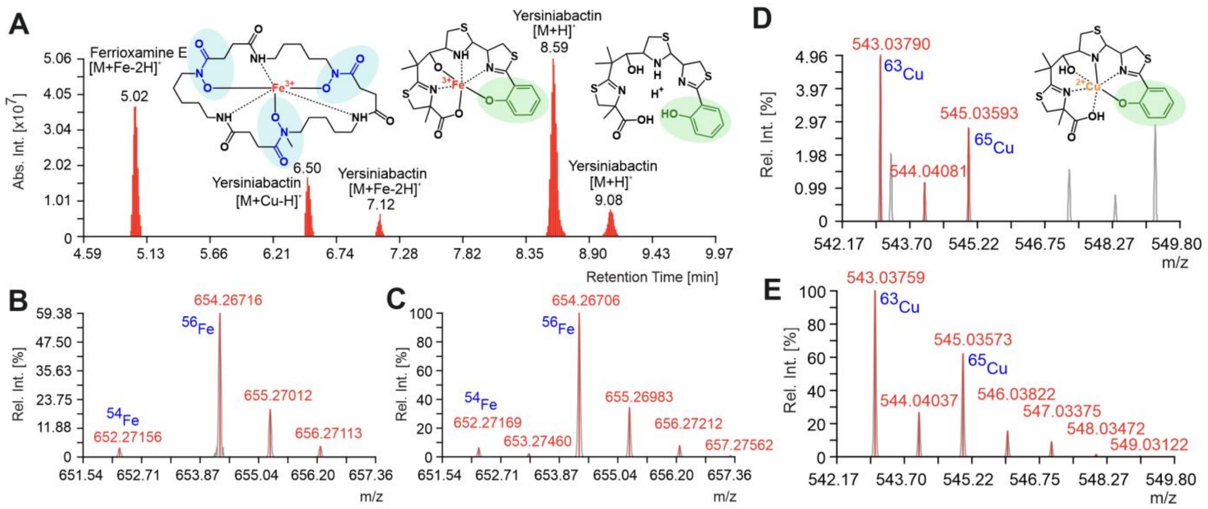

Figure S1. Yersiniabactin profiling in *Klebsiella* spp. Reconstructed ion profiles of Ybt isoforms **(A)** are suggested by CycloBranch software for a urine sample received from female patient (No. 11, **Table S1**) treated for AP. Ferrioxamine E was used as an internal standard and provided characteristic iron isotopic cluster **(B)** with an excellent match to the theoretical one **(C)**. The recorded cupric Yer isotope structures **(D)** matched the theoretical isotopic envelope **(E)**. Blue or the green shaded areas in siderophore structures illustrate hydroxamate and phenolate groups, respectively.

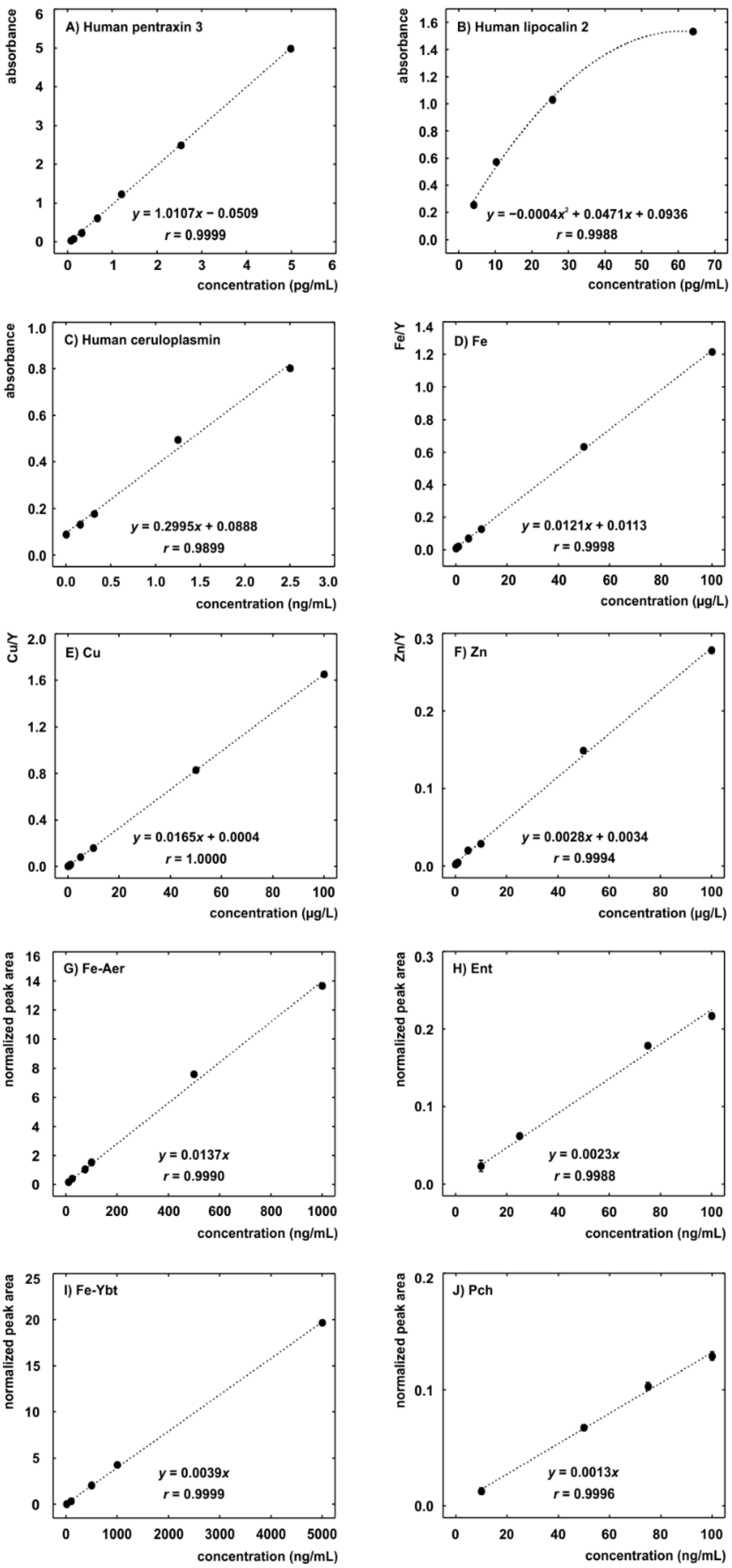

Figure S2: Calibration curves of Ptx3, Lcn2, Cp, total Fe, total Zn, total Cu, Fe-Aer, Ent, Fe-Ybt, and Pch. A) Human Pentraxin-3; LOD 0.022 pg/mL; LOQ 0.066 pg/mL; r=0.9999. **B)** Human Lipocalin-2; LOD 1.0 pg/mL; LOQ 5.0 pg/mL; r=0.9988. **C)** Human ceruloplasmin; LOD 6.1 pg/mL;

LOQ 29.4 pg/mL; r=0.9899. **D)** Total Fe; LOD 1.3 µg/L=2.81 µg/mmol; LOQ 2.6 µg/L=5.49 µg/mmol; r=0.9998. **E)** Total Cu; LOD 0.3 µg/L=0.67 µg/mmol; LOQ 0.7 µg/L=1.59 µg/mmol; r=1.0000. **F)** Total Zn; LOD 1.6 µg/L=3.34 µg/mmol; LOQ 3.7 µg/L=7.88 µg/mmol; r=0.9994. **G)** Fe-Aer; LOD 1.5 ng/mL; LOQ 4.5 ng/mL; r=0.9990. **H)** Ent; LOD 3.1 ng/mL; LOQ 9.3 ng/mL; r=0.9988. **I)** Fe-Ybt; LOD 2.2 ng/mL; LOQ 6.7 ng/mL; r=0.9999. **J)** Pch; LOD 1.8 ng/mL; LOQ 5.5 ng/mL; r=0.9996. Average creatinine value: 9.291 mmol/L.

Determination of total copper, iron and zinc levels in urine by ICP-MS

Quantitation was performed using eight-point external calibration. The total metal concentration levels were normalised to creatinine and expressed in µg/mmol. Urine samples were measured in six replicates. The instrumental limits of detection (LODs) a instrumental limits of quantitation (LOQs) are shown in Figure S1 Independently prepared quality control samples (10 µg/L) were measured periodically to ensure the reliability of the quantitative results.

Sample Preparation and LC-MS Analysis

The standard ferri-Aer (Fe-Aer) was obtained through custom-based synthesis, and the LC-MS provided three forms (desferri-Aer, anhydro-desferri-Aer, and Fe-Aer), which were considered in the calibrations. Desferri-enterobactin (Ent), desferri pyochelin (Pch), and ferri-Ybt (Fe-Ybt) were obtained from EMC Microcollections (Tübingen, Germany), and were considered in quantitation. Urine samples (50 µL) were spiked with the ferriform of the internal standard, ferrioxamine E (FoxE, 2 µg/mL, 5 µL), to reach a final concentration of 100 ng/mL. Subsequently, the mixture was extracted using pre-cooled isopropanol (400 µL) at a temperature of –80 °C and stored in a deep freezer for one hour. Following centrifugation at 10,000 rpm for 10 minutes at 4 degrees Celsius, the protein-free supernatants were transferred to new 0.5-mL vials and the solvents were evaporated under reduced pressure for 60 minutes at 35 degrees Celsius. Prior to LC-MS analysis, samples were resuspended in 100 µL of 5% acetonitrile (Acn). Calibration points and urine samples were measured in six and two technical replicates, respectively. The analytes were separated at a flow rate of 50 µL/min using a gradient elution with a 13-minute program. The mobile phase consisted of 0.1% formic acid in 1% Acn (solvent A) and 99% Acn (solvent B). The gradient elution was conducted as follows: 2% B (0–1 minute), 99% B (1–9 minute), 99% B (9–10 minute), 2% B (10–10.1 minute), and 2% B (10.1–13 minute). Mass spectra were collected in the electrospray ionization positive-ion mode within the 100–1500 Da spectral range, with mass accuracy better than 1 ppm in most cases. The sustainability of the LC-MS system was evaluated on a regular basis using the Peptide Standard Mixture (Sigma-Aldrich, MO, USA) and continuous online calibration with the ESI Tuning Mix for Ion Traps (Sigma-Aldrich, MO, USA). Metallophores were classified according to their precise molecular weights, isotope patterns, and retention times. Extracted ion chromatograms with a spectral width of 0.005 Da were integrated, summed, and normalized to the response of the ferrioxamine E (FoxE) ferriform.The urine samples were deemed to exhibit metallophore positivity if the concentration of at least one urine Cr-indexed bacterial metabolite exceeded the LOD for a specific marker as defined by liquid chromatography-mass spectrometry.

Table S1: Cohort inpatients admitted for the treatment of AP and cUTI. Pt, patient; Cr: urinary creatinine; Cu/Cr: total copper index; Fe/Cr: total iron index; Zn/Cr: total zinc index; Cp/Cr: ceruloplasmin index; Lcn2/Cr: Lipocalin-2 index; Ptx3/Cr: Pentraxin-3 index;Fe-Aer/Cr: ferriform of aerobactin index ; Fe-Ybt/Cr: ferriform of yersiniabactin index; Cu-Ybt/Cr: copper form of yersiniabactin index; Pch/Cr: pyochelin index; Ent/Cr: enterobactin index; ND: not detected; DET: detected (a value between LOD and LOQ); n.a.: not available; *E. coli*: *Escherichia coli*; *E. cloacae*: *Enterococcus cloacae*; *S. aureus*: *Staphylococcus aureus*; *K. pneumoniae*: *Klebsiella pneumoniae*. Metal, metallophore and protein concentrations were normalized to Cr and expressed in µg/mmoL or ng/mmoL.

Pt	Sex	Age	AP / cUTI	Cr [mmol/L]	Microbiology	Cu/Cr [µg/mmoL]	Fe/Cr [µg/mmoL]	Zn/Cr [µg/mmoL]	Cp/Cr [µg/mmoL]	Lcn2/Cr [µg/mmoL]	Ptx3/Cr [ng/mmoL]	(Fe)-Aer/Cr [µg/mmoL]	Ybt/Cr [µg/mmoL]	Fe-Ybt/Cr [µg/mmoL]	Cu-Ybt/Cr [µg/mmoL]	Pch/Cr [µg/mmoL]	Ent/Cr [µg/mmoL]	Antibiotic treatment
1	m	60	AP	29.1	*S. aureus*	5.2	2.0	56.8	9.5	1.9	24.3	ND	ND	ND	ND	ND	ND	No
2	f	38	AP	11.3	*E. coli*	3.8	DET	44.3	45.1	1.6	67.5	10.336	ND	ND	ND	ND	ND	No
3	f	78	AP	10.0	negative	7.4	10.1	50.2	77.5	0.45	43	ND	ND	ND	ND	ND	ND	Yes
4	f	40	AP	5.9	*E. coli*	DET	DET	38.5	22.9	2.0	38.6	ND	ND	ND	ND	ND	ND	No
6	f	43	AP	5.3	negative	3.3	205.1	151.1	113.9	1.5	37.5	ND	ND	ND	ND	ND	ND	Yes
7	f	62	AP	8.4	*K. pneumoniae*	22.3	75.2	229.3	191.0	2.8	20.1	53.735	ND	ND	ND	ND	ND	No
8	f	54	AP	3.0	*Klebsiella* spp.	28.6	1933.9	275.0	180.8	3.9	117.5	ND	ND	ND	ND	ND	ND	Yes
9	f	40	AP	4.4	*P. mirabilis*	14.2	1133.8	81.8	20.3	3.7	n.a.	ND	ND	ND	ND	ND	ND	No
10	m	65	AP	6.0	*K. pneumoniae*	11.8	39.0	145.9	439.8	0.45	84	ND	ND	ND	ND	ND	ND	Yes
11	f	65	AP	18.8	*Klebsiella* spp.	2.4	15.2	96.4	64.9	5.7	71	ND	56.036	4.478	10.414	ND	ND	Yes
12	f	45	AP	11.0	*Klebsiella* spp.	4.8	10.5	411.2	83.4	1.9	19	ND	ND	ND	ND	ND	ND	No
15	f	19	AP	4.5	*E. coli haemolytica*	DET	15.9	ND	22.4	2.5	32.1	11.313	ND	ND	ND	ND	ND	No
17	f	49	AP	7.8	*E. coli haemolytica*	13.8	179.8	55.7	33.5	0.45	32.7	ND	ND	ND	ND	ND	ND	Yes
18	m	40	AP	14.4	*E. coli*	5.7	7.4	23.4	96.4	0.45	7.1	2.410	ND	ND	ND	ND	ND	No
20	f	18	AP	13.8	*E. coli*	2.0	74.0	57.5	13.7	0.45	15	ND	ND	ND	ND	ND	ND	No
21	f	26	AP	18.7	*E. coli haemolytica*	DET	9.5	7.1	11.7	0.45	15.3	ND	ND	ND	ND	ND	ND	No
22	f	53	AP	9.9	*E. coli*	8.5	8.7	65.5	16.6	0.45	26.8	ND	ND	ND	ND	ND	ND	Yes
5	m	54	cUTI	11.3	*Enterobacter* spp.	2.4	DET	55.7	8.0	0.1	ND	ND	ND	ND	ND	5.508	ND	No
13	m	71	cUTI	12.3	*E. coli haemolytica*	4.5	13.9	108.7	113.3	0.45	234.7	11.416	ND	ND	ND	ND	ND	Yes
14	f	63	cUTI	3.4	*E. cloacae*	12.2	21.2	47.9	1201.0	10.6	979.1	3.769	ND	ND	2.617	ND	ND	No
16	m	81	cUTI	13.1	*E. coli haemolytica*	1.7	32.6	104.6	9.8	0.45	6.9	ND	ND	ND	ND	ND	ND	No
19	f	72	cUTI	18.3	*E. coli haemolytica*	3.5	4.1	62.5	44.9	11.5	25.6	ND	3.979	ND	ND	ND	ND	No
23	m	77	cUTI	6.9	*S. aureus*	6.0	14.2	93.5	41.6	1.0	38.3	ND	ND	ND	ND	ND	ND	No

Table S2: Diagnostic accuracy of single level tests. The controls were compared either with separate AP-cUTI or AC groups or with the (AP-cUTI + AC) sum. AP-cUTI: acute pyelonephritis-complicated urinary tract infection; AC: acute cystitis; Ctrl: controls; Cu/Cr: total copper index; Fe/Cr: total iron index; Zn/Cr: total zinc index; Cp/Cr: ceruloplasmin index; Lcn2/Cr: Lipocalin-2 index; Ptx3/Cr: Pentraxin-3 index; LC-MS: liquid chromatography-mass spectrometry; TP: true positive; TN: true negative; FN: false negative; FP: false positive. The cut-off values refer to a test specificity of 90%. Metal and protein concentrations were normalised to creatinine.

**Study group**	**Sensitivity and Specificity**	**Cu/Cr cut-off: 4.8 µg/mmoL**	**Cp/Cr cut-off: 7.9 µg/mmoL**	**Lcn2/Cr cut-off: 0.1 µg/mmoL**	**LC-MS cut-off: positive detection**	**Ptx3 cut-off: 9.8 ng/mmoL**
**AP-cUTI**	TP	15	23	22	9	19
	FN	8	0	1	14	3
**AC**	TP	9	22	24	5	18
	FN	19	6	4	23	9
**Ctrl**	FP	5	4	1	0	4
	TN	39	40	43	44	38
**Sensitivity and specificity of biomarker panels**						
**AP-cUTI**	Sensitivity	65%	100%	96%	39%	86%
	Specificity	89%	91%	98%	100%	90%
**AC**	Sensitivity	32%	79%	86%	18%	67%
	Specificity	89%	91%	98%	100%	90%
**AP-cUTI + AC**	Sensitivity	47%	88%	90%	27%	76%
	Specificity	89%	91%	98%	100%	90%

Table S3: Control cohort inpatients admitted for elective surgery and healthy volunteers. Cr: urinary creatinine; Cu/Cr: total copper index; Fe/Cr: total iron index; Zn/Cr: total zinc index; Cp/Cr: ceruloplasmin index; Lcn2/Cr: Lipocalin-2 index; Ptx3/Cr: Pentraxin-3 index; ND: not detected; DET: detected (a value between LOD and LOQ); n.a.: not available. Microbiology and metallophore detection were negative in all cases. Metal and protein concentrations were normalised to Cr and are expressed in µg/mmoL or ng/mmoL. All microbiology analyses were negative in the control cohort.

Patient	Sex	Age	Cr [mmol/L]	Cu/Cr [µg/mmoL]	Fe/Cr [µg/mmoL]	Zn/Cr [µg/mmoL]	Cp/Cr [µg/mmoL]	Lcn2/Cr [µg/mmoL]	Ptx3/Cr [ng/mmoL]	Metallophores [µg/mmoL]
1	f	91	4.9	1.8	DET	78.3	7.6	ND	DET	ND
2	m	32	18.3	3.3	20.0	40.5	2.5	ND	13.3	ND
3	m	42	7.9	DET	DET	34.5	DET	ND	17.5	ND
4	m	58	19.7	1.8	10.0	61.1	4.8	0.0	9.9	ND
5	m	71	7.1	DET	99.2	58.2	DET	ND	DET	ND
6	m	32	18.3	1.9	59.4	40.2	1.8	ND	6.6	ND
7	m	75	8.4	DET	27.7	62.3	DET	ND	DET	ND
8	m	19	28.3	1.0	25.0	31.8	ND	ND	5.7	ND
9	m	67	12.0	2.4	95.9	97.0	3.0	0.0	ND	ND
10	m	49	8.4	4.1	154.7	82.0	ND	0.6	9.7	ND
11	m	72	8.2	2.4	20.7	41.2	DET	ND	ND	ND
12	m	80	8.2	6.2	11.3	29.0	DET	0.0	ND	ND
13	m	61	9.3	DET	20.9	130.1	ND	ND	n.a.	ND
14	m	72	9.3	1.6	95.8	57.9	ND	ND	ND	ND
15	m	38	17.5	0.9	9.1	42.6	ND	0.0	ND	ND
16	m	82	6.7	DET	32.4	60.6	ND	ND	ND	ND
17	m	68	13.4	1.1	4.9	110.5	ND	ND	ND	ND
18	m	82	9.5	DET	11.5	56.5	ND	ND	ND	ND
19	m	46	4.2	ND	ND	86.4	ND	ND	ND	ND
20	m	71	4.2	DET	17.2	76.9	0.7	ND	ND	ND
21	m	54	6.0	8.3	1138.6	70.1	ND	ND	DET	ND
22	m	68	8.2	2.4	272.8	42.8	8.5	0.1	ND	ND
23	m	75	5.0	DET	94.3	63.2	DET	ND	ND	ND
24	m	59	9.4	2.0	143.8	28.9	ND	ND	9.4	ND

25	m	73	9.1	DET	DET	9.8	DET	ND	ND	ND
26	f	66	11.6	2.3	DET	29.6	ND	ND	ND	ND
27	m	65	24.0	1.5	3.0	28.8	ND	0.1	DET	ND
28	m	82	5.6	3.8	12.4	49.8	ND	ND	DET	ND
29	m	28	24.5	0.7	4.4	27.2	ND	ND	ND	ND
30	m	62	7.8	4.6	702.6	27.1	ND	ND	6.4	ND
31	m	47	26.4	0.9	22.0	32.1	ND	ND	12.2	ND
32	m	69	11.4	DET	ND	75.8	ND	ND	ND	ND
33	m	66	7.0	DET	ND	66.8	ND	ND	ND	ND
34	m	80	5.9	DET	ND	49.3	DET	ND	ND	ND
35	m	74	13.1	2.2	ND	106.0	ND	ND	9.3	ND
36	m	68	7.3	DET	ND	169.3	5.4	ND	DET	ND
37	m	43	1.6	DET	134.8	DET	18.4	ND	ND	ND
38	f	38	5.3	DET	41.2	DET	ND	ND	ND	ND
39	f	42	4.9	DET	50.4	ND	6.0	ND	n.a.	ND
40	f	49	2.3	ND	DET	DET	12.8	ND	ND	ND
41	f	59	9.2	DET	5.8	25.0	DET	ND	DET	ND
42	m	27	23.5	1.0	DET	22.1	29.9	ND	8.0	ND
43	m	44	4.5	DET	42.4	DET	1.4	ND	ND	ND
44	f	49	5.1	DET	DET	41.5	ND	ND	DET	ND

Table S4: Cohort outpatients seeking treatment for AC. Cr: urinary creatinine; Cu/Cr: total copper index; Fe/Cr: total iron index; Zn/Cr: total zinc index; Cp/Cr: ceruloplasmin index; Lcn2/Cr: Lipocalin-2 index; Ptx3/Cr: Pentraxin-3 index; Fe-Aer/Cr: ferriform of aerobactin index; Fe-Ybt/Cr: ferriform of yersiniabactin index; Cu-Ybt/Cr: copper form of yersiniabactin index; Pch/Cr: pyochelin index; Ent/Cr: enterobactin index; ND: not detected; DET: detected (a value between LOD and LOQ); n.a.: not available; *E. coli*: *Escherichia coli*; *S. saprophyticus*: *Staphylococcus saprophyticus*; *S. marcescens*: *Serratia marcescens*; *C. albicans*: *Candida albicans*; *P. aeruginosa*: *Pseudomonas aeruginosa.* Metal, metalophore and protein concentrations were normalized to Cr.

Patient	Sex	Age	Cr [mmol/L]	Microbiology	Cu/Cr [µg/mmoL]	Fe/Cr [µg/mmoL]	Zn/Cr [µg/mmoL]	Cp/Cr [µg/mmoL]	Lcn2/Cr [µg/mmoL]	Ptx3/Cr [ng/mmoL]	(Fe)-Aer/Cr [µg/mmoL]	Ybt/Cr [µg/mmoL]	Fe-Ybt/Cr [µg/mmoL]	Cu-Ybt/Cr [µg/mmoL]	Pch/Cr [µg/mmoL]	Ent/Cr [µg/mmoL]
1	f	71	8.8	*Klebsiella spp.*	2.3	DET	24.5	9.4	ND	7.2	ND	ND	ND	ND	ND	ND
2	f	42	9.3	*E. coli*	3.7	13.8	19.8	51.4	DET	ND	ND	ND	ND	ND	ND	ND
3	f	30	11.5	*S. saprophyticus*	2.2	41.0	63.8	16.9	DET	63.5	ND	ND	ND	ND	ND	ND
4	f	30	2.4	*S. saprophyticus*	17.0	DET	64.0	27.7	0.3	ND	ND	ND	ND	ND	ND	ND
5	f	67	3.4	*E. coli*	DET	79.5	65.5	DET	6.7	48.8	ND	ND	ND	ND	ND	ND
6	f	32	28.4	*E. coli haemolytica*	0.9	2.9	11.7	12.7	DET	n.a.	ND	ND	ND	ND	ND	ND
7	f	55	8.5	*E. coli haemolytica*	6.9	347.1	22.4	124.7	DET	68.7	ND	ND	24.497	5.714	ND	ND
8	f	46	4.6	*E. coli*	3.7	16.4	40.3	65.5	7.0	39.3	ND	ND	ND	ND	ND	ND
9	f	51	3.7	*E. coli*	7.1	DET	68.4	27.9	9.9	44.2	ND	ND	ND	ND	ND	ND
10	f	40	8.4	*E. coli*	6.5	423.8	54.9	5.9	2.6	37.2	ND	ND	ND	ND	ND	ND
11	m	37	13.9	*E. coli haemolytica*	4.0	165.4	133.8	44.5	DET	8.7	ND	ND	2.505	ND	ND	ND
12	f	34	3.1	*E. coli*	8.7	803.2	89.3	DET	ND	31.6	ND	ND	ND	ND	ND	ND
13	f	27	5.3	*E. coli haemolytica*	3.4	168.0	63.3	63.9	DET	53.1	ND	ND	ND	ND	ND	ND
14	f	31	8.1	*S. saprophyticus*	DET	116.5	23.5	9.2	DET	16	ND	ND	ND	ND	ND	ND
15	f	88	7.7	*E. coli haemolytica*	9.4	19.8	76.4	202.5	0.8	671.6	ND	1.872	6.395	4.787	ND	ND
16	f	20	22.4	*S. saprophyticus*	3.0	56.9	20.8	13.8	DET	22.3	ND	ND	ND	ND	ND	ND
17	f	22	8.0	*E. coli haemolytica*	2.5	98.6	27.0	7.2	4.3	ND	5.031	ND	ND	ND	ND	ND
18	f	51	16.9	*S. marcescens*	1.3	DET	35.3	8.8	DET	13.4	ND	ND	ND	ND	ND	ND
19	f	24	6.7	*E. coli*	2.3	13.6	50.3	45.8	DET	24.9	ND	ND	ND	ND	ND	ND
20	f	20	1.1	*E. coli*	ND	DET	248.8	72.8	ND	ND	ND	ND	ND	ND	ND	ND
21	f	21	14.9	*E. coli haemolytica*	1.9	9.9	46.2	52.9	DET	4.3	ND	ND	ND	ND	ND	ND
22	f	48	1.3	*E. coli*	DET	65.3	520.9	27.0	DET	58.5	ND	ND	ND	ND	ND	ND
23	m	77	18.5	*C. albicans*	2.1	10.0	95.7	11.3	0.3	40.6	ND	ND	ND	ND	ND	ND
24	f	34	1.4	*E. coli haemolytica*	DET	106.3	201.1	37.0	0.3	ND	ND	ND	ND	ND	ND	ND
25	f	23	7.0	*S. saprophyticus*	3.1	12.1	70.3	15.2	3.7	17.4	ND	ND	ND	ND	ND	ND
26	f	30	2.5	*E. coli haemolytica*	DET	617.2	340.6	217.9	DET	227.8	ND	ND	ND	23.514	ND	ND
27	f	77	20.8	*E. coli*	1.2	ND	70.9	2.5	ND	4.3	ND	ND	ND	ND	ND	ND
28	m	83	18.9	*P. aeruginosa*	1.1	2.8	88.3	5.7	0.3	88.7	ND	ND	ND	ND	ND	ND

Table S5: Diagnostic accuracy in a combination test. TP: true positive; FN: false negative; FP: false positive, TN: true negative; LOD: limit of detection; Cr: urinary creatinine, Cu/Cr; total copper index; Lcn2/Cr: Lipocalin-2 index; LC-MS: liquid chromatography-mass spectrometry. Acute pyelonephritis (AP) and complicated urinary tract infections (cUTI) were considered as positive finding. Acute cystitis (AC) was considered as negative finding. Metal and protein concentrations were normalised to creatinine. Urine samples with a concentration of Lcn2/Cr ≤ 0.45 µg/mmoL were considered for the combination test (pink/orange shading).

**Results of biomarker analyses**				**Criteria for a combination test**		
**Urine sample**	**Lcn2/Cr (µg/mmol)**	**Cu/Cr (µg/mmol)**	**LC-MS detection**	**cut-off: Lcn2/Cr 0.45 µg/mmoL**	**1st cut-off: Lcn2/Cr 0.45 µg/mmoL<br><br>2nd cut-off: Cu/Cr 4.8 µg/mmoL**	**1st cut-off: Lcn2/Cr 0.45 µg/mmoL<br><br>2nd cut-off: Cu/Cr 4.8 µg/mmoL *OR* LC-MS positive detection**
**AP and cUTI patient cohort**						
1	1.9	5.2	negative	TP	TP	TP
2	1.6	3.8	positive	TP	TP	TP
3	0.45	7.4	negative	FN	TP	TP
4	2.0	1.5	negative	TP	TP	TP
5	0.1	2.4	positive	FN	FN	TP
6	1.5	3.3	negative	TP	TP	TP
7	2.8	22.3	positive	TP	TP	TP
8	3.9	28.6	negative	TP	TP	TP
9	3.7	14.2	negative	TP	TP	TP
10	0.45	11.8	negative	FN	TP	TP
11	5.7	2.4	positive	TP	TP	TP
12	1.9	4.8	negative	TP	TP	TP
13	0.45	4.5	positive	FN	FN	TP
14	10.6	12.2	positive	TP	TP	TP
15	2.5	1.5	positive	TP	TP	TP
16	0.45	1.7	negative	FN	FN	FN
17	0.45	13.8	negative	FN	TP	TP
18	0.45	5.7	positive	FN	TP	TP
19	11.5	3.5	positive	TP	TP	TP

20	0.45	2.0	negative	FN	FN	FN
21	0.45	1.6	negative	FN	FN	FN
22	0.45	8.5	negative	FN	TP	TP
23	1.0	6.0	negative	TP	TP	TP
**AC patient cohort**						
1	0.10	2.3	negative	TN	TN	TN
2	0.45	3.7	negative	TN	TN	TN
3	0.45	2.2	negative	TN	TN	TN
4	0.3	17.0	negative	TN	TN	FP
5	6.7	1.5	negative	FP	FP	FP
6	0.45	0.9	negative	TN	TN	TN
7	0.45	6.9	positive	TN	FP	FP
8	7.0	3.7	negative	FP	FP	FP
9	9.9	7.1	negative	FP	FP	FP
10	2.6	6.5	negative	FP	FP	FP
11	0.45	4.0	positive	TN	TN	FP
12	0.10	8.7	negative	TN	TN	FP
13	0.45	3.4	negative	TN	TN	TN
14	0.45	1.5	negative	TN	TN	TN
15	0.8	9.4	positive	FP	FP	FP
16	0.45	3.0	negative	TN	TN	TN
17	4.3	2.5	positive	FP	FP	FP
18	0.45	1.3	negative	TN	TN	TN
19	0.45	2.3	negative	TN	TN	TN
20	0.10	0.6	negative	TN	TN	TN
21	0.45	1.9	negative	TN	TN	TN
22	0.45	1.5	negative	TN	TN	TN
23	0.3	2.1	negative	TN	TN	TN
24	0.3	1.5	negative	TN	TN	TN
25	3.7	3.1	negative	FP	FP	FP
26	0.45	1.5	positive	TN	TN	FP
27	0.10	1.2	negative	TN	TN	TN
28	0.3	1.1	negative	TN	TN	TN

**Sensitivity and specificity of the combination tests**				
	**TP**	13	18	20
	**FN**	10	5	3
	**TN**	21	15	16
	**FP**	7	8	12
	**Sensitivity**	57%	78%	87%
	**Specificity**	75%	65%	57%
